# Exploring the Ability of Meningococcal Vaccines to Elicit Mucosal Immunity: Insights from Humans and Mice

**DOI:** 10.3390/pathogens10070906

**Published:** 2021-07-18

**Authors:** Elissa G. Currie, Scott D. Gray-Owen

**Affiliations:** Department of Molecular Genetics, Temerty Faculty of Medicine, University of Toronto, Toronto, ON M5S 1A8, Canada; elissa.currie@mail.utoronto.ca

**Keywords:** *Neisseria meningitidis*, meningococcus, nasal infection, sepsis, vaccine, mucosal immunity, herd immunity, 4CMenB, humanized mouse model

## Abstract

*Neisseria meningitidis* causes a devastating invasive disease but is also a normal colonizer of the human nasopharynx. Due to the rapid progression of disease, the best tool to protect individuals against meningococcal infections is immunization. Clinical experience with polysaccharide conjugate vaccines has revealed that an ideal meningococcal vaccine must prevent both invasive disease and nasal colonization, which confers herd immunity. However, not all meningococcal vaccines are equal in their ability to prevent nasal colonization, for unknown reasons. Herein, we describe recent efforts to utilize humanized mouse models to understand the impact of different meningococcal vaccines on nasal colonization. These mice are susceptible to nasal colonization, and they become immune following live nasal infection or immunization with matched capsule-conjugate or protein-based vaccines, replicating findings from human work. We bring together insights regarding meningococcal colonization and immunity from clinical work with findings using humanized mouse models, providing new perspective into the different determinants of mucosal versus systemic immunity. Then, we use this as a framework to help focus future studies toward understanding key mechanistic aspects left unresolved, including the bacterial factors required for colonization and immune evasion, determinants of nasal mucosal protection, and characteristics of an ideal meningococcal vaccine.

## 1. Introduction

*Neisseria meningitidis* (the meningococcus) is a Gram-negative bacterial pathogen that is an obligate colonizer of the human nasopharynx. Nasal colonization is asymptomatic in nature; however, under rare circumstances, *N. meningitidis* can penetrate mucosal tissues to cause severe invasive disease [1]. Invasive meningococcal disease most commonly presents as meningitis and sepsis, but may also cause gastrointestinal symptoms, septic arthritis, pericarditis, and invasive pneumoniae [2,3]. If left untreated, invasive meningococcal disease is lethal in upward of 50% of patients [4]. Despite the availability of effective antibiotic treatment options, fatality rates remain above 10%, with a large percentage of survivors experiencing serious lifelong morbidities [5,6]. The most effective way to reduce the burden of invasive meningococcal disease is through immunization, and much effort has been devoted toward the development of meningococcal vaccines.

The most successful meningococcal vaccines currently in use are those that use capsule polysaccharides conjugated to a protein carrier as the vaccine antigen [4]. *N. meningitidis* serogroups are defined on the basis of capsule polysaccharides to give a total of 13 serogroups, of which six (A, B, C, W, X, and Y) are responsible for the vast majority of invasive meningococcal disease [4]. Vaccines using capsule polysaccharides are available for serogroups A, C, W, and Y. Polysaccharide conjugate vaccines are extremely successful at preventing invasive disease by the respective serogroups in vaccinated individuals, and they have the added effect of preventing *N. meningitidis* nasal colonization; this has been particularly evident following immunization with capsule-conjugate vaccines targeting serogroup A and those targeting serogroup C [7,8,9,10,11,12,13,14]. Prevention of nasal colonization reduces the transmission of vaccine serogroups through a vaccinated population, thus reducing the risk of invasive disease in unvaccinated or otherwise nonimmune individuals. This immunity to nasal colonization is exemplified in the reduced nasal burden observed during carriage studies, as well as reduced invasive disease documented in unvaccinated individuals. The indirect protection against invasive disease afforded to unvaccinated individuals is referred to as herd immunity [15,16].

Following the success of conjugate vaccines in controlling meningococcal disease through the induction of herd immunity, prevention of nasal colonization is now considered the gold standard to which all future meningococcal vaccines strive [15]. Unfortunately, the immune processes that confer protection against meningococcal nasal colonization are poorly understood, making it difficult to target these processes during vaccine design. This challenge is exacerbated because *N. meningitidis* does not naturally colonize the nose of any organism other than humans, which hampers understanding of processes related to nasal colonization, as well as preclinical evaluation of vaccines. Without an animal model or an accepted correlate of protection against nasal colonization, meningococcal vaccines have been approved without any predicted effect on mucosal immunity. Impact on nasal colonization is, therefore, only appreciated after vaccine implementation, through large clinical studies and immunization campaigns.

Herein, we describe advances to our understanding of meningococcal colonization through use of humanized mouse models, considering parallels with data from human studies and with emphasis on aspects that can inform vaccine design and testing. Specifically, we consider the relative contribution of classical correlates of immune protection against invasive meningococcal disease, including serum bactericidal assays, versus other effector mechanisms that contribute to mucosal protection. Key in this regard, we highlight the distinction between immune responses elicited by nasal colonization versus parenteral immunization, and we consider differences in the protection afforded by polysaccharide capsule-conjugate versus protein-based vaccines. Lastly, we consider how the mouse-based models can be used to complement findings from human surveillance and vaccine studies to better understand the mucosal lifestyle of *N. meningitidis* and its complex interplay with the various immune effector mechanisms.

## 2. CEACAM1-Humanized Mice as a Model for Meningococcal Nasal Colonization

Wild-type mice are not colonized by *N. meningitidis*. The introduction of a transgene encoding the human carcinoembryonic antigen-related cell adhesion molecule 1 (hCEACAM1) renders mice susceptible to nasal colonization by *N. meningitidis* [17,18,19]. These transgenic mice, herein referred to as hCEACAM1 mice, present a useful tool to shed light onto the meningococcal lifestyle within the mucosa and host–pathogen interactions that occur during meningococcal nasal infection.

While mice express CEACAM1, *Neisseria* do not bind the murine ortholog. Expression of the hCEACAM1 transgene mirrors the pattern seen in human tissues and, importantly, is expressed in the nasopharynx where it can be accessed by *N. meningitidis* during nasal infection [17,19]. Colonization is quantified by the number of colony-forming units (CFU) recovered from the mouse nose at various time points following infection [17]. A diverse array of meningococcal strains are capable of colonizing hCEACAM1 mice, including historically relevant lab strains [17,20], as well as low-passage clinical strains [21]. *N. meningitidis* binds to hCEACAM1 via its colony opacity-associated (Opa) proteins [17]. Colonization of hCEACAM1 mice is strictly dependent on the interaction between neisserial Opa proteins and mouse hCEACAM1 expression. Opa expression is controlled by phase variation in *N. meningitidis* and is, therefore, turned randomly ‘on’ and ‘off’ during bacterial growth and division. Bacteria that are genetically *opa*-deficient fail to colonize the mice [17]. Furthermore, when an inoculum was prepared with only phase variants that had Opa expression turned ‘off’, all bacteria recovered from mice were expressing Opa proteins, thus reflecting an in vivo selection for Opa expression [17]. This finding parallels studies done in human volunteers with *Neisseria gonorrhoeae*, wherein an inoculum was prepared with Opa expression turned ‘off’, but all bacteria recovered from the urethra of male volunteers were Opa-expressing [22]. Thus, the importance of the interaction between Opa and hCEACAM1 demonstrated in the mouse model is reflective of an important interaction during human infection.

Colonization of hCEACAM1 mice is variable for different strains in terms of rate of colonization (number of mice colonized) and burden of colonization (CFU recovered per mouse). Some strains tested will colonize upward of 90% of infected mice, while others will colonize as few as 20–30% of mice ([17,20,21] and unpublished data). Colonization is also short-term, with most transgenic mice clearing nasal infection within 10–14 days post inoculation, corresponding with the time required for the emergence of an adaptive immune response [17]. In humans, colonization can be chronic and last up to a year [23]. This short-term colonization in mice is likely reflective of the extreme human restricted nature of *N. meningitidis*, given that the meningococcus binds only the human forms of various host proteins, including those for iron acquisition and complement evasion. The addition of other human factors may increase utility of this model in studying chronic infections. However, in its current form, the hCEACAM1 model still presents an opportunity to study many factors surrounding meningococcal colonization in a living organism, including the innate and adaptive immune responses to infection, as well as testing the efficacy of drugs, immunotherapies, and vaccines.

## 3. Serum Bactericidal Antibodies and Protection from Invasive Disease

Pathogen-specific antibodies can drive bacterial clearance from a host through a number of different mechanisms. *N. meningitidis*-specific antibodies that activate the complement system through the classical complement cascade are known as serum bactericidal antibodies (SBA), and they are key in protection against invasive meningococcal disease [4,15,24]. The first observation of a link between anti-*N. meningitidis* SBA titer and protection against invasive disease was reported in the 1960s [25], and it has been recapitulated over the years. SBA titers are now accepted as a correlate of protection against invasive meningococcal disease, and elicitation of SBA is used as a correlate of protection against invasive disease [25,26,27,28,29,30]. The contribution of SBA to protection against nasal colonization is not well understood; however, recent experiences with protein-based meningococcal vaccines (discussed below) suggests that serum SBA titers do not predict protection against nasal colonization.

## 4. Infection-Induced Immunity to Nasal Colonization

Rates of *N. meningitidis* nasal colonization increase through childhood and peak in young adults in industrialized nations [31]. It is generally assumed that, prior to the introduction of vaccines, the majority of young adults would have been colonized intermittently throughout their adolescence. Naturally occurring nasal infection with *N. meningitidis* induces an antibody response in humans that is detectable in serum and saliva [25,27,32,33,34]. Antibody titers increase with age, corresponding with increased rates of colonization [34]. Ex vivo studies using human tonsil tissues have revealed the presence of *N. meningitidis*-reactive B and T cells, suggesting that nasal colonization induces the maturation of a local adaptive immune response [35,36]. These studies have been instrumental in increasing our understanding of immune response elicited by the meningococcus in its natural host during its natural state of infection, within the nasal mucosa.

Similar to observations made in humans, nasal infection of hCEACAM1 mice induced an anti-meningococcal antibody response [17,20]. Infection-experienced mice exhibit nasal anti-meningococcal IgA and serum anti-meningococcal IgG, reflecting the salivary and serum antibody responses observed in humans [17,20,33,34]. An advantage of a mouse model is that the infection history of mice can be controlled. Mice are truly naïve to *N. meningitidis* at the time of infection, thus allowing for the study of the immune response to each infection, as well as the cross-reactivity of this immune response with heterologous strains. Infection-induced immunity in hCEACAM1 mice requires two nasal infections in order to induce immunity to a subsequent nasal challenge [17]. This suggests that a single nasal exposure to *N. meningitidis* is insufficient to drive immunity to subsequent infections in a mouse model of colonization. It is notable that repeated nasal administration of killed *N. meningitidis* does not develop sterilizing immunity, indicating that prolonged carriage or other activities of the viable meningococci elicit this response. It is possible that a single chronic infection, as expected to naturally occur in humans, would be sufficient to confer immunity to subsequent colonization, or it may be possible that natural immunity is indeed reliant on repeat nasal exposures throughout an individual’s lifetime [23].

While nasal infection induced a marked local (nasal mucosal) IgA response in the hCEACAM1 mice, it also elicited the production of serum antibodies that exhibited cross-reactivity with heterologous strains of *N. meningitidis* [20]. Interestingly, antibody cross-reactivity was not limited to strains of the same serogroup of the challenge strain nor to those expressing the same families of immunodominant antigens (such as outer membrane porins) [20]. This suggests that the antibodies induced through nasal infection target diverse antigens on the meningococcus, beyond just the capsule polysaccharides and immunodominant outer membrane porins. Infection-induced immunity to nasal colonization was also cross-protective. Infection-experienced mice were protected from nasal colonization with strains expressing the same capsule type or other major antigens, but not from distantly related strains for which no cross-reactive antibody was detected [20]. This cross-reactive immune response in mice suggests that a human would acquire immunity to numerous strains of *N. meningitidis* following sustained colonization with a single strain, but exposure to a variety of strains would be necessary to induce immunity to the majority of circulating meningococcal isolates. This is important, given that the meningococcal species is highly genetically diverse [37].

The mouse model for meningococcal nasal colonization presents a novel opportunity to study natural infection-induced immunity in a number of ways. One exciting avenue would be to expand on the information gained from studies using human tonsil tissues to look at meningococcal-specific immune cells in local mucosal tissues, as well as in systemic compartments such as the bone marrow, spleen, and thymus. A key question is whether mucosal *N. meningitidis*-specific immune cells, such as those observed in human tonsils, are required for immunity against nasal colonization and how they contribute to protection. Within the mouse model, a kinetic analysis of lymphocyte recruitment and activation can be explored, and genetic or other approaches can be used to demonstrate their contribution to immunity. This would reveal the determinants of mucosal protection and provide clarity regarding the breadth of cross-protection afforded by infection.

## 5. Polysaccharide Conjugate Vaccines

Polysaccharide conjugate vaccines are capable of inducing protection against meningococcal nasal colonization in humans [7,8,9,10,11,12,13,14]. Plain polysaccharides are T-cell-independent antigens that are poorly immunogenic in children and do not induce long-lasting immunity in adults [38]. The covalent linkage of polysaccharides to a protein carrier converts polysaccharides into T-cell-dependent antigens, which results in increased antibody titers post vaccination that are sustained for longer periods of time, most likely due to T helper cells facilitating B-cell maturation [39,40]. However, for historical reasons, the protein carriers used in currently licensed meningococcal conjugate vaccines include either the chemically inactivated tetanus or diphtheria toxoids, or an inactivate mutant of the diphtheria toxin, CRM197, none of which are expressed by *N. meningitidis* [4]. The protection afforded by these conjugate vaccines will, therefore, be restricted to B cell, plasma cell, and antibody responses to the capsular polysaccharide, since the protein-specific responses are irrelevant to the meningococci. However, parenteral immunization does not elicit mucosal IgA, which is classically attributed to mucosal protection. Given that little is known regarding the effector mechanisms of systemically produced IgG within the nasal mucosa, the relative contribution of complement, opsonophagocytic, or other processes to protection against meningococcal colonization remains unknown.

As noted previously, vaccine-elicited antibodies that induce complement-dependent killing, as measured by SBA, are instrumental in protection against invasive meningococcal infections and are currently used as a correlate of protection during vaccine development [15]. While polysaccharide conjugate vaccines induce robust SBA titers in serum [41], antibody opsonization leading to phagocytic killing of the meningococcus has also been reported as an important mechanism of clearance of *N. meningitidis* infections [42,43]. Moreover, protection against colonization by other bacterial pathogens has been attributed to antibody-dependent bacterial agglutination [44,45]. Notably in this regard, immunization of hCEACAM1 mice with the serogroup C capsule-conjugate vaccine protected against nasal colonization [17], recapitulating the protection observed in humans. This provides an opportunity to evaluate how anti-capsular antibodies confer immunity against nasal colonization in these mice, and to test whether these processes could be promoted through different vaccination formulations and/or routes of administration.

## 6. Protein Vaccines

The majority of invasive meningococcal infections in Europe and North America are caused by serogroup B *N. meningitidis* [46]. While polysaccharide-based vaccines have effectively targeted select serogroups of *N. meningitidis*, the serogroup B capsule polysaccharides mimic human antigens and are, therefore, unsuitable for use as a vaccine component [47]. Two vaccines, 4CMenB and rLP2086, are currently approved for the prevention of serogroup B *N. meningitidis* [48,49,50]. This review focuses on the impact of 4CMenB immunization since these have been tested against meningococcal colonization in the CEACAM1-humanized mice.

The 4CMenB vaccine, which contains OMVs and three recombinant proteins, was developed to target serogroup B *N. meningitidis* [48,49,51,52]. The impact of this vaccine on invasive disease was predicted using SBAs and the meningococcal antigen typing systems (MATs) [53,54,55,56]. These antibody assays predict that 4CMenB immunization should protect immunized individuals against the majority of circulating serogroup B strains in England and Canada [55,56,57]. However, 4CMenB was approved without any ability to predict its impact on nasal colonization. Population studies are ongoing; however, current data suggest that mass 4CMenB immunization does not impact nasal colonization rates [58,59,60,61].

A difficulty with interpreting the impact of 4CMenB immunization on nasal colonization is that this vaccine is a subcapsular vaccine. Unlike experience with capsule polysaccharide vaccines, implementation of 4CMenB is not expected to completely abrogate invasive disease caused by serogroup B strains because the targeted antigens vary in sequence and expression level [62]. However, while 4CMenB was developed with specific focus on serogroup B meningococcal strains, immunization can also impact strains that are not serogroup B due to shared antigens [62]. This means that monitoring total serogroup B nasal colonization rates may not capture the total impact of 4CMenB immunization.

To evaluate the impact of 4CMenB experimentally, hCEACAM1 mice were immunized twice with 4CMenB and challenged with a sepsis model of invasive meningococcal disease or via nasal infection. As expected, based on antibody titers, immunization with 4CMenB was protective against invasive disease by all strains tested [21]. Notably, immunization also protected against invasive challenge by the serogroup W strain Bufa, which expresses 4CMenB vaccine antigen NadA. This experimentally supports the ability of subcapsular vaccines to prevent disease caused by different serogroups.

In contrast to the clear effect on sepsis, 4CMenB immunization did not confer protection against nasal colonization by approximately half of the strains tested [21]. This suggests that antibody responses, while being reliable predictions of protection against invasive disease, do not predict protection against colonization. This finding is in agreement with clinical studies, where immunized individuals are protected against invasive disease, but no observable impact on colonization or herd immunity has been documented [58,59,60,61].

It is instructive that 4CMenB immunization did confer protection against colonization by some of the challenge isolates. Immunization prevented colonization by strain NZ98/254, which matches vaccine antigens PorA and fHbp and is the source of the vaccine’s OMV preparation, and by two out of the three tested low-passage clinical isolates, which matched vaccine antigens PorA, NHBA, and fHbp or PorA and fHbp, respectively [21]. Prevention of colonization by strains that express numerous vaccine antigen matches may suggest that mucosal immunity elicited by 4CMenB requires a high density of targeted epitopes on the bacterial surface, either due to high-level expression or reactivity against multiple antigens. Experimental validation of this point may allow the potential impact of this and other protein-based vaccines on community carriage to be more effectively modeled.

## 7. Interaction between Human Protein and Immunizing Antigen

By virtue of the fact that protein-based vaccines tend to target essential and surface-expressed virulence factors, most meningococcal protein vaccine antigens interact directly with human proteins. An example of this is factor H-binding protein (fHbp), which is an antigen in both 4CMenB and rLP2086. FHbp complexes directly with human, but not mouse, factor H, to decorate the bacterial cell surface with this complement regulatory protein [42,63]. Factor H is a negative regulator of complement; thus, this serves to protect the bacteria from complement-mediated killing [63,64]. Antibodies binding to fHbp can block the recruitment of factor H to the bacterial surface, thus decreasing bacterial resistance to serum, while also facilitating bacterial killing in an antibody-dependent manner [65]. The advantage to the bacteria afforded by factor H binding, as well as the importance of blocking this interaction, is not captured in a wild-type or hCEACAM1 mouse model, but can be recapitulated in a mouse that expresses transgenic human factor H [66].

The recombinant meningococcal fHbp included in 4CMenB is proposed to bind to human factor H during immunization [67]. This interaction between fHbp and human factor H can shield antigen epitopes from the immune system, as well as reduce the immunogenicity of the vaccine antigen. Indeed, when human factor H-expressing transgenic mice or rhesus macaques (which also express factor H recognized by fHbp) were immunized with intact fHbp, they exhibited lower anti-meningococcal antibody titers than did animals immunized with a factor H binding-defective fHbp mutant [67,68,69]. These data clearly suggest that interaction between an antigen and a host protein upon immunization can be detrimental to vaccine efficacy.

While the fHbp-based studies to date have focused on the relative efficacy of mutant antigens in the context of invasive disease, double transgenic mice, expressing both hCEACAM1 and human factor H, will be required to model both the positive and the negative impacts of immunization with fHbp during mucosal colonization. A similar problem of antigen complexing with host proteins during immunization could occur when using other immunizing antigens and, hence, must be considered during future vaccine development. Indeed, a binding-defective mutant of the bacterial transferrin receptor is more immunogenic and protective than the binding-competent parental form when used as a vaccine immunogen, implying that its complexing with the host iron-binding protein transferrin also interferes with the adaptive response [70]. Notable in this regard, human transferrin is functionally expressed by transgenic mouse lines such that these animals are highly susceptible to invasive infection by *N. meningitidis*, facilitating testing of vaccine efficacy against invasive disease and allowing comparisons of virulence differences between meningococcal strains [71,72]. Mice co-expressing hCEACAM1 with human transferrin will provide a means to understand the contribution of transferrin receptors to bacterial growth within the mucosa, as well as understand how their complexing with the host-derived transferrin subverts the immune response. The use of additional transgenes or supplements, such as the exogenous administration of human serum proteins, will be required when considering the relative efficacy of vaccines that target other human-restricted virulence factors.

## 8. Outstanding Questions and Future Directions

While the invasive phases of disease cause the devastating consequences of meningococcal infection, these necessarily follow nasopharyngeal colonization. Thus, effective mucosal immunity will protect the immune individual while also providing herd protection. Despite its importance, the lifestyle of meningococci within mucosal tissues remains poorly understood, and the determinants of sterilizing immunity remain undefined. Exemplifying this point, a number of factors have been associated with increasing an individual’s risk of *N. meningitidis* nasal carriage, including smoking in British teenagers [73], the dry season in the meningitis belt in Africa [74,75], and previous influenza infection [76], yet a mechanistic explanation for these associations remains unknown. While mouse models cannot replicate all aspects of infection, judicious development and application of new models can allow direct questions such as these to be addressed. Similarly, mechanistic studies comparing the relative efficacy of different immunization strategies can shed light onto what immune processes confer mucosal protection, and how to improve cross-protection so as to provide broad-spectrum coverage against all meningococcal strains. Mouse-based studies with other human upper respiratory tract pathogens, including *Streptococcus pneumoniae* [77,78,79,80] and *Bordetella pertussis* [81,82,83,84], highlight the utility of this approach by revealing an unexpected contribution of T lymphocytes as the effectors governing nasal protection. Thus, by combining bacterial and mouse genetics with drug and immune-based interventions, the humanized mouse models can test hypotheses and provide new insight regarding the specific contribution of putative virulence factors to infection and disease, to understand the fine balance between immunity and immunopathogenesis, and to reveal where rational vaccine design may further enhance protection.

## Data Availability

Not applicable.
